# Additional lateral extra-articular tenodesis in revision ACL reconstruction does not influence the outcome of patients with low-grade anterior knee laxity

**DOI:** 10.1007/s00402-021-04145-y

**Published:** 2021-08-29

**Authors:** Lena Eggeling, T. C. Drenck, J. Frings, M. Krause, Alexander Korthaus, Anna Krukenberg, Karl-Heinz Frosch, Ralph Akoto

**Affiliations:** 1 Asklepios Clinic St. Georg, Lohmühlenstraße 5, 20099 Hamburg, Germany; 2 Department of Trauma and Orthopaedic Surgery, Sports Traumatology, BG Hospital Hamburg, Bergedorfer Str. 10, 21033 Hamburg, Germany; 3 Department of Trauma and Orthopaedic Surgery, University Medical Center Hamburg-Eppendorf, Martinistrasse 52, 20246 Hamburg, Germany; 4 Cologne Merheim Medical Center, University of Witten/Herdecke, Cologne, Germany

**Keywords:** Revision ACLR, LET, Indication, Low-grade anterior knee instability

## Abstract

**Introduction:**

There is limited evidence on the indications of lateral extra-articular tenodesis (LET) in revision ACLR. The aim of this study was to evaluate the influence of the LET in patients with revision ACLR with preoperative low-grade anterior knee laxity.

**Methods:**

Between 2013 and 2018, 78 patients who underwent revision ACLR with preoperative low-grade anterior knee laxity [≤ 5 mm side-to-side difference (SSD)] were included in the retrospective cohort study. An additional modified Lemaire tenodesis was performed in 23 patients during revision ACLR and patients were clinically examined with a minimum of 2 years after revision surgery. Postoperative failure of the revision ACLR was defined as SSD in Rolimeter^®^ testing ≥ 5 mm or pivot-shift grade 2/3.

**Results:**

In total, failure of the revision ACLR occurred in 11.5% (*n* = 9) of the cases at a mean follow-up of 28.7 ± 8.8 (24–67) months. Patients with an additional LET and revision ACLR did not show a significantly reduced failure rate (13% vs. 11%) or an improved clinical outcome according to the postoperative functional scores or pain in regards to patients with an isolated revision ACLR (Tegner 5.7 ± 1.3 vs. 5.9 ± 1.5, n.s.; IKDC 77.5 ± 16.2 vs. 80.1 ± 14.9, n.s., Lysholm 81.9 ± 14.2 vs. 83.8 ± 14.5, n.s.; VAS 1.9 ± 2.2 vs. 1.2 ± 1.7, n.s.).

**Conclusions:**

An additional LET in patients with revision ACLR with low-grade anterior knee laxity does not influence patient-related outcomes or failure rates. Subjects with preoperative low-grade anterior knee laxity may not benefit from a LET in revision ACLR.

**Level of evidence:**

III

## Introduction

Adjunctive anterolateral extra-articular reconstructions reduce rotational instability and re-rupture rates in anterior cruciate ligament (ACL) surgery and lead to good patient-related outcomes, reduces excessive internal rotation and restricts laxity [1–7]. Authors have revealed that combined revision anterior cruciate ligament reconstruction (ACLR) and lateral extra-articular tenodesis (LET) improve clinical and radiological outcomes [8, 9]. Previous studies demonstrated that an additional LET in patients with primary ACLR and high-grade anterior knee laxity improves patient-related outcome and reduces the risk that an ACLR will fail [10–13]. According to the literature, the indication for an additional LET is revision ACLR, high-grade pivot-shift, generalised ligamentous laxity/genu recuvatum and young patients returning to pivoting activities [14, 15]. However, potential complications with LET procedures are joint overconstraint and loss of range of motion [16, 17].

Especially for revision ACLR, the indication for additional LET seems to be generous in the current literature [14], although the data for revision in contrast to primary ACLR is significantly lower [18]. Studies that directly compare revision ACLR with and without LET are currently rare [15, 19]. A previous study showed a significantly lower failure rate with an additional LET for revision ACLR and high-grade knee laxity (side-to-side difference ≥ 6 mm, pivot-shift grade 3) [15]. In revision ACLR, it has not yet been clarified which patients will benefit from an additional LET. However, to the best of our knowledge, the effect of a LET in patients with low-grade anterior knee laxity (side-to-side difference ≤ 5 mm, pivot-shift grade 1–2) and revision ACLR has not been examined before.

Hence, the aim of this study was to evaluate the influence of the LET in patients with revision ACLR with preoperative low-grade anterior knee laxity. We hypothesized that an additional LET will not influence the clinical outcome of revision ACLR in this low-risk patient population.

## Materials and methods

### Patient population

Between 2013 and 2018, 198 patients underwent revision ACLR at our institution. From the year 2015 to 2016, an additional LET was performed standardly in revision ACLR, since 2017 an additional LET was performed in case of high-grade knee laxity. All procedures were performed by two experienced surgeons (R.A. and K-H.F.).

**Fig. 1:**
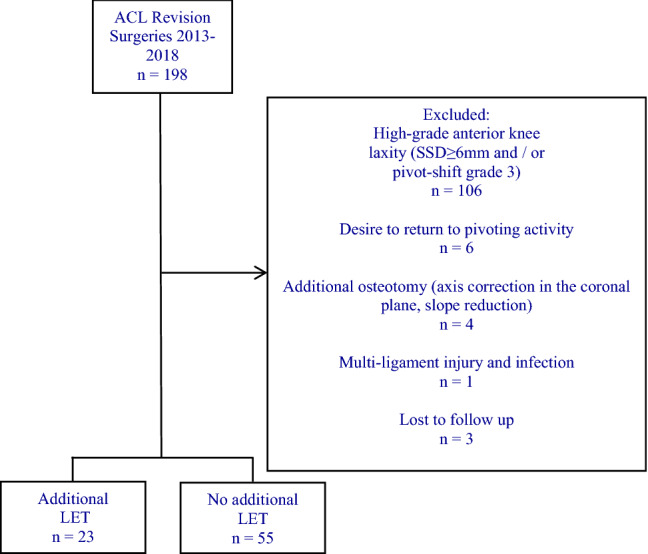
A flow-chart of the inclusion and exclusion criteria

Inclusion criteria were persistent or recurrent instability after ACLR at the time of revision surgery with preoperative low-grade anterior knee laxity (defined as side-to-side difference (SSD) ≤ 5 mm in Rolimeter^®^ testing and pivot-shift grade 1 and 2 in examination under anaesthesia) and written informed consent to participate in the study. Exclusion criteria were the desire to return to pivoting activities (soccer, basketball, etc., *n* = 6), high-grade anterior knee laxity (defined as SSD ≥ 6 mm in Rolimeter^®^ testing or/and pivot-shift grade 3 in examination under anaesthesia, *n* = 106), additional osteotomy (axis correction in the coronal plane, slope reduction, *n* = 4), multi-ligament injury and infection (*n* = 1) and loss to follow- up (*n* = 3) (Fig. 1).

The study design was approved by the local Ethics Committee and an informed consent was obtained by each patient (PV5590).

Seventy-eight patients with a follow-up of 28.7 ± 8.8 (mean ± SD; range 24–67) were included in the retrospective cohort study and examined with a minimum follow-up of two years. The patients were contacted at least two years after revision ACLR by telephone, and after they provided consent to participate in the study, they were invited for an examination. An isolated revision ACLR was performed in 55 patients. An additional LET (modified Lemaire tenodesis) was carried out in 23 patients.

Clinical testing protocol before revision ACLR and at the time of follow-up obtained the Lysholm and Tegner Score [20, 21]. The knee laxity was assessed pre- and postoperatively by the Lachman test, pivot-shift test and Rolimeter^®^. The pivot-shift test was divided into grade 1 (glide), grade 2 (clunk) and grade 3 (gross) and the Lachman test was rated with the 2000 Knee Examination Form from the International Knee Documentation Committee (IKDC) (Grade 1: 2–5 mm, grade 2: 6–10 mm and grade 3: > 10 mm) [22]. At the time of follow-up additionally the subjective IKDC form and the Knee Injury and Osteoarthritis Outcome Score (KOOS) were recorded [23, 24]. The Lysholm score was devided into excellent (95–100), good (84–94), fair (65–83) and poor (< 65). Subjective pain was quantified using the visual analogous scale (VAS) [25]. Failed revision ACLR was defined as SSD in Rolimeter^®^ testing ≥ 5 mm or pivot-shift grade 2/3 [26]).

### Surgical technique for revision ACLR

A single-bundle revision ACLR was performed in anterolateral portal technique. The graft choice depended on the previous harvested tendons and hamstrings, bone-patellar tendon-bone or quadriceps grafts were preferred.

**Fig. 2:**
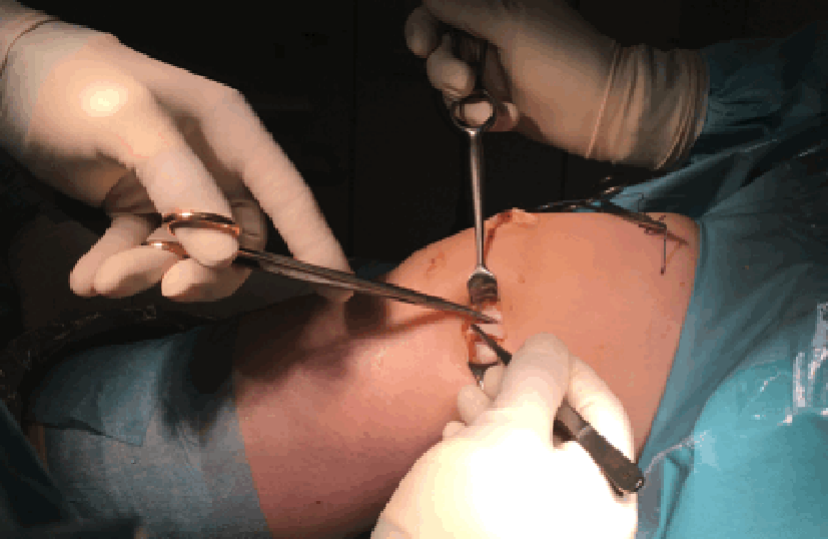
A strip of the distal ‘tractus iliotibialis’ with connection to the Gerdy tubercle was dissected

**Fig. 3:**
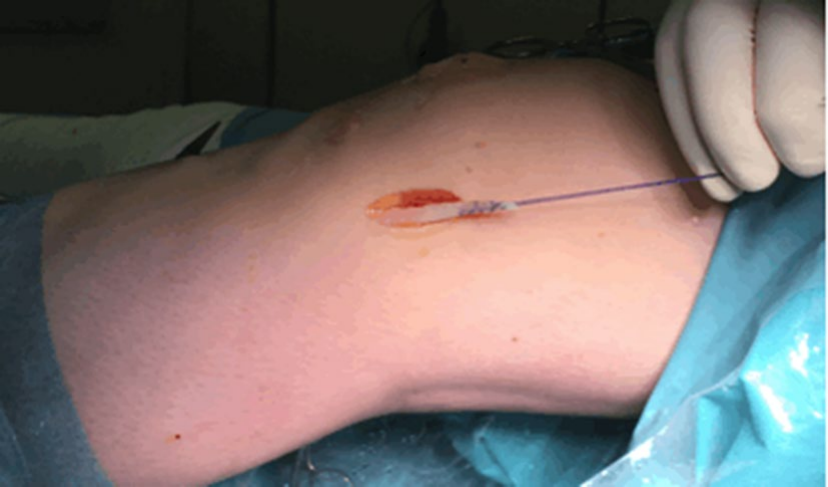
The strip of the distal ‘tractus iliotibialis’ was secured with a Vicryl suture

**Fig. 4:**
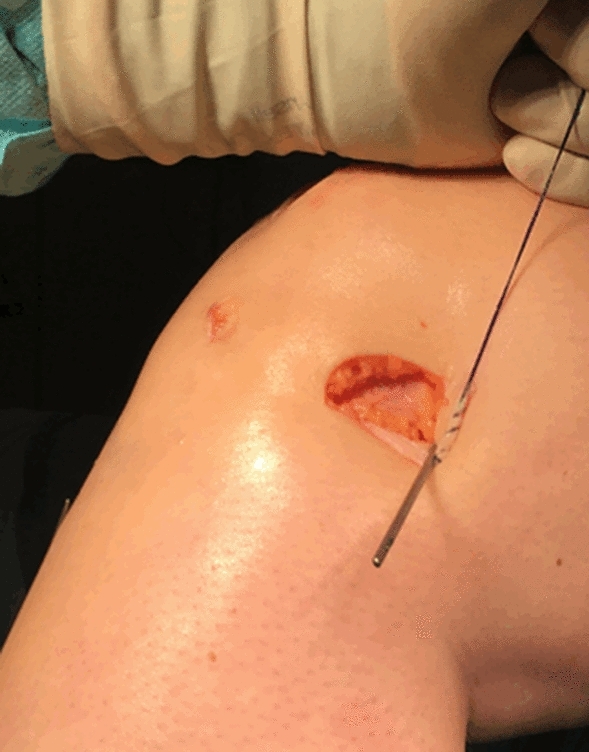
The k-wire was attached 1 cm proximal and posterior to the lateral epicondyle

**Fig. 5:**
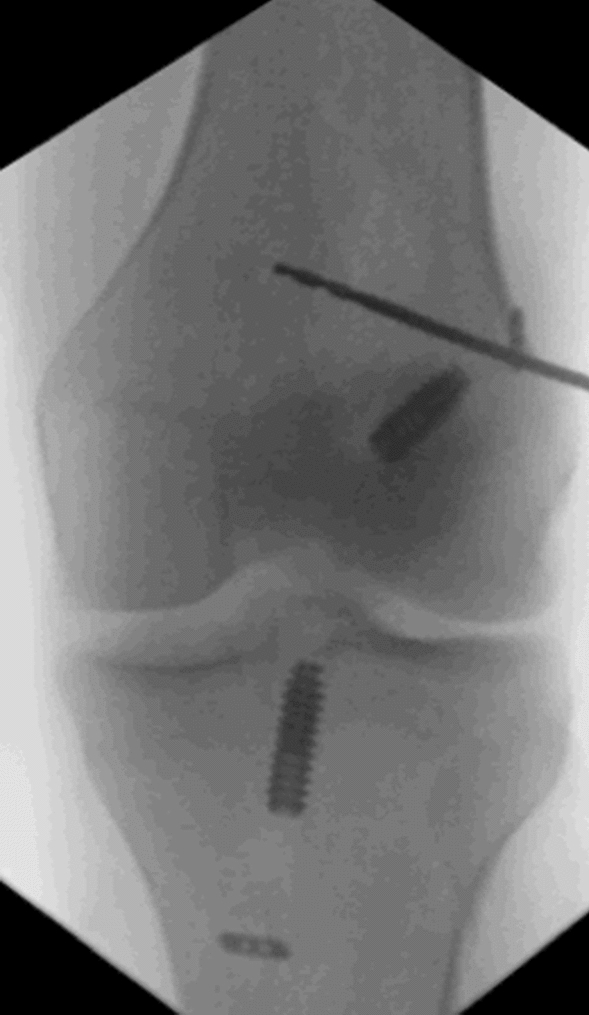
X-ray of the knee with the k-wire placed 1 cm proximal and posterior to the lateral epicondyle

An adjunctive LET, using the modified Lemaire technique, was carried out in 23 patients after we introduced the LET in revision ACLR. In the area of the lateral epicondyle, an approximately 4 cm skin incision was made (Fig. 2). A strip of the distal ‘tractus iliotibialis’ (6–8 cm long and 6–8 mm wide) with connection to the Gerdy tubercle was dissected. The strip was secured with a Vicryl suture (Fig. 3) and attached 1 cm proximal and posterior to the lateral epicondyle (Figs. 4 and 5) via a 5 mm tunnel and an interference screw at 45° flexion [27].

### Statistical analysis

Statistical analysis was performed using IBM^®^ SPSS^®^ Statistics Version 25. For continuous variables the mean ± standard deviation was used. The calculation was based on two groups: patients with isolated revision ACLR or additional LET and revision ACLR. A subgroup analysis was performed to compare patients with pivot-shift grade 2 between the two groups. Differences between the groups were calculated with the Student’s *t* test and the Kruskal–Wallis test for non-parametric parameters. Categorical parameters were compared using the chi-squared test and the Fisher’s exact text was used for categorical parameters in case of small subgroups (*n* < 5). A *p* value less than 0.05 was considered significant.

## Results

Overall, there were 78 patients (males = 48, females = 30) with revision ACLR with low-grade anterior knee laxity with a mean age at revision surgery of 32.3 ± 10.6 (16–55) years. There were no differences between an isolated or combined LET and ACLR regarding age, gender, side of the knee, body mass index and further characteristics (Table 1).

**Table 1:** Patient characteristics according to the isolated revision ACLR and combined LET + revision ACLR (*n* = 78)

Characteristics	Isolated revision ACLR (*n* = 55)	LET + revision ACLR (*n* = 23)	*p* value
Female Sex, *n* (%)	20 (36.4)	10 (43.5)	0.556
Left knee, *n* (%)	24 (43.6)	13 (56.5)	0.299
Age at the time of revision ACLR, mean ± SD (minimum–maximum)	31.9 ± 9.9 (16–52)	33.3 ± 12.3 (16–55)	0.616
Body mass index (BMI) > 30 kg/m^2^, *n* (%)	8 (14.5)	5 (21.7)	0.437
Traumatic mechanism of graft failure preoperatively, *n* (%)	35 (63.6)	10 (43.5)	0.100
Number of previous ACL procedures, *n* (%)			
1	47 (85.5)	16 (69.6)	0.314
2	6 (10.9)	5 (21.7)	
3	1 (1.8)	1 (4.3)	
4	1 (1.8)	1 (4.3)	

*SD* standard deviation*, ACL* anterior cruciate ligament, *ACLR* ACL reconstruction

Preoperatively, there was no difference between the two groups according to the Lachman test. A pivot-shift grade 2 occurred significantly more often in patients with an additional LET (74% vs. 35%, *p* = 0.005). Preoperative scores like VAS, Tegner and Lysholm Score showed no significant difference between the two groups. The radiological findings (coronal alignment, osteoarthritis or preoperative femoral tunnel malposition) did not differ in the compared groups of isolated or combined LET and revision ACLR (Table 2).

**Table 2:** Preoperative clinical and radiological findings of revision ACLR according to the isolated and combined LET + ACLR (*n* = 78)

Characteristics	Isolated revision ACLR (*n* = 55)	LET + revision ALCR (*n* = 23)	*p* value
Grade of preoperative Lachman test, *n* (%)			
Grade 1 (2–5 mm)	24 (43.6)	6 (26.1)	0.146
Grade 2 (5–10 mm)	31 (56.4)	17 (73.9)	
Grade of preoperative pivot shift, *n* (%)			
Grade 0	5 (9.1)	0	0.005
Grade 1	31 (56.4)	6 (26.1)	
Grade 2	19 (34.5)	17 (73.9)	
Preoperative VAS, mean in points ± SD (minimum—maximum)	4.2 ± 2.2 (0–10)	4.3 ± 2.9 (0–9)	0.855
Preoperative Tegner rating system, mean in points ± SD (minimum—maximum)	3.1 ± 1.5 (0–6)	2.6 ± 1.6 (0–5)	0.211
Preoperative Lysholm Score, mean in points ± SD (minimum—maximum)	52.7 ± 22.3 (3–100)	49.2 ± 28.1 (3–100)	0.779
Coronal alignment, *n* (%)			0.110
Valgus malalignment	1 (1.8)	3 (13)	
Varus malalignment	4 (7.3)	1 (4.3)	
Osteoarthritis, *n* (%)	19 (34.5)	9 (39.1)	0.700
Preoperative femoral tunnel malposition, *n* (%)	13 (23.6)	5 (21.7)	0.302

*ACLR* anterior cruciate ligament reconstruction, *VAS* visual analogeous scale, *SD* standard deviation

A subgroup analysis of patients with a preoperative pivot-shift grade 2 showed no difference in failure rate, VAS pain, pre and postoperative Lysholm, Tegner and IKDC score according to the two groups (failure rate: 10.9% vs. 13%, n.s., VAS 1.2 ± 1.7 vs. 1.9 ± 2.2, n.s., Lysholm 83.8 ± 14.5 vs. 81.9 ± 14.2, n.s.; Tegner 5.9 ± 1.5 vs. 5.7 ± 1.3 n.s.; IKDC 80.1 ± 14.9 vs. 77.5 ± 16.2, n.s., Table 3).

**Table 3:** Failure rate and postoperative functional outcome in patients with pivot-shift grade 2 in regards to the isolated and combined LET + revision ACLR (*n* = 36)

Patients with pivot-shift grade 2	Isolated revision ACLR (*n* = 19)	LET + revision ALCR (*n* = 17)	*p* value
Failure rate of the revision ACLR, *n* (%)	3 (15.8)	3 (17.6)	0.614
Preoperative VAS, mean in points ± SD (minimum–maximum)	3.4 ± 2.1 (0–8)	4.1 ± 3 (0–9)	0.616
Preoperative Tegner rating system, mean in points ± SD (minimum–maximum)	3 ± 1.5 (0–5)	2.2 ± 1.5 (0–5)	0.137
Preoperative Lysholm Score, mean in points ± SD (minimum–maximum)	49.6 ± 20.1 (3–70)	47.2 ± 27.7 (3–90)	0.138
Postoperative VAS, mean in points ± SD (minimum–maximum)	1.4 ± 2.1 (0–7)	1.6 ± 1.9 (0–6)	0.510
Postoperative Tegner rating system, mean in points ± SD (minimum–maximum)	6 ± 1.7 (3–9)	5.8 ± 1.8 (3–8)	0.651
Postoperative Lysholm Score, mean in points ± SD (minimum–maximum)	83 ± 16.6 (41–100)	83.8 ± 13.6 (58–100)	0.882
Postoperative subjective IKDC score, mean in points ± SD (minimum–maximum)	79.2 ± 17.7 (32–95)	79.4 ± 15.5 (53–100)	0.900

*ACLR* anterior cruciate ligament reconstruction, *VAS* visual analogous scale, *IKDC* International Knee Documentation Committee, *SD* standard deviation

In total, 68% of the patients (*n* = 53) reported a good to excellent clinical outcome and 32% (*n* = 25) reported a fair to poor outcome. Failure occurred in 11.5% of the cases (*n* = 9). Patients with an additional LET did not show a significantly reduced failure rate (13% vs. 11%) or improved clinical outcome according to the postoperative functional scores (Tegner, Lysholm, IKDC and KOOS) or pain (VAS) in regards to patients with an isolated ACLR (Table 4). There was no significant difference in postoperative knee laxity (Lachman and pivot-shift test). The SSD in Rolimeter testing in the group of LET and ACLR was 1.3 ± 2 mm whereas patients with an isolated ACLR showed a SSD of 1.8 ± 2.1 mm but the analysis did not show a statistically significant difference between the two groups. In the group of combined LET and revision ACLR 17.4% of the patients (*n* = 4) complained about pain in the area of the LET when resting or moving. There was no statistical significant difference regarding return to sports between the groups (Table 4).

**Table 4:** Postoperative clinical and radiological findings of revision ACLR according to the isolated and combined LET + ACLR (*n* = 78)

Characteristics	Isolated revision ACLR (*n* = 55)	LET + revision ALCR (*n* = 23)	*p* value
Grade of postoperative Lachman test, *n* (%)			
Grade 0 (< 2 mm)	46 (83.6)	18 (78.3)	0.701
Grade 1 (2–5 mm)	6 (10.9)	3 (13)	
Grade 2 (5–10 mm)	3 (5.5)	2 (8.7)	
Grade of postoperative pivot shift, *n* (%)			
Grade 0	46 (83.6)	20 (87)	0.934
Grade 1	3 (5.5)	1 (4.3)	
Grade 2	6 (10.9)	2 (8.7)	
Rolimeter testing postoperatively, mean in mm ± SD (minimum–maximum)	1.8 ± 2.1 (0–10)	1.3 ± 2 (0–7)	0.121
PTS postoperatively, mean in ° ± SD (minimum–maximum)	9.6 ± 1.9 (5–15)	9.9 ± 2.3 (7–16)	0.532
Postoperative tenderness on palpation of the knee, *n* (%)	11 (20)	6 (26.1)	0.553
Postoperative VAS, mean in points ± SD (minimum–maximum)	1.2 ± 1.7 (0–7)	1.9 ± 2.2 (0–6)	0.142
Postoperative Tegner rating system, mean in points ± SD (minimum–maximum)	5.9 ± 1.5 (3–9)	5.7 ± 1.3 (3–8)	0.577
Postoperative Lysholm Score, mean in points ± SD (minimum–maximum)	83.8 ± 14.5 (41–100)	81.9 ± 14.2 (57–100)	0.493
Postoperative subjective IKDC score, mean in points ± SD (minimum–maximum)	80.1 ± 14.9 (32–95)	77.5 ± 16.2 (41–100)	0.479
KOOS postoperative, mean in points ± SD (minimum–maximum)			
Symptoms	87.3 ± 14.8 (50–100)	87.6 ± 15.4 (57–100)	0.943
Pain	87.9 ± 14.1 (39–100)	87.9 ± 14.6 (42–100)	0.992
Function, daily living	93 ± 10 (60–100)	95.2 ± 8.2 (66–100)	0.613
Function, sports and recreational activities	76 ± 22.7 (5–100)	72.6 ± 25.9 (20–100)	0.682
Quality of life	58.4 ± 19.7 (6–88)	63.8 ± 18.9 (31–94)	0.245
Failed revision ACLR, *n* (%)	6 (10.9)	3 (13)	0.530
Return to sports, *n* (%)	24 (43.6)	11 (47.8)	0.734

*ACLR* anterior cruciate ligament reconstruction, *PTS* posterior tibial slope *SD* standard deviation, *VAS* visual analogous scale, *IKDC* International Knee Documentation Committee, *KOOS* Knee Injury and Osteoarthritis Outcome Score

The analysis revealed that a total of 44.9% of the patients (*n* = 35) had a medial meniscus lesion and 20.5% of patients (*n* = 16) had a lateral meniscus lesion at the time of revision surgery. The choice of revision ACLR graft was also not significantly different between an isolated or combined ACLR and LET (Table 5).

**Table 5:** Surgical details of patients with isolated revision ACLR and combined LET + revision ACLR (*n* = 78)

Characteristics	Isolated revision ACLR (*n* = 55)	LET + revision ACLR (*n* = 23)	*p* value
Choice of revision ACLR graft, *n* (%)			0.221
Patellar-bone-tendon-bone	31 (56.4)	7 (30.4)	
Hamstring tendon	13 (23.6)	9 (39.1)	
Quadriceps tendon	11 (20)	7 (30.4)	
Medial meniscal lesion in total, *n* (%)	24 (43.6)	11 (47.8)	0.805
Medial meniscus repair, *n* (%)	8 (14.5)	4 (17.4)	
Partial medial meniscus resection, *n* (%)	15 (27.3)	6 (26.1)	
Total medial meniscus resection, *n* (%)	1 (1.8)	1 (4.3)	
Lateral meniscal lesion in total, *n* (%)	12 (21.8)	4 (17.4)	0.766
Lateral meniscus repair, *n* (%)	4 (7.3)	1 (4.3)	
Partial lateral meniscus resection, *n* (%)	8 (14.5)	3 (13)	

*SD* standard deviation*, ACLR* anterior cruciate ligament reconstruction

## Discussion

The main finding of this study was that an additional LET did not influence the outcome of patients who underwent revision ACLR with preoperative low-grade anterior knee laxity. Beyond that, an adjunctive LET did not reduce failure rates or improve postoperative functional scores in this low-risk patient population.

Previous studies indicate that additional anterolateral procedures will reduce the risk of failure and improve patient-related outcomes in revision ACLR [7, 19, 28].

Redler et al. have shown that a combined revision ACLR with a doubled gracilis and semitendinosus autograft and LET in 118 patients led to a significant clinical improvement with a mean follow-up of 10.6 years [8], while Ferretti et al. demonstrated good to very good results with combined LET and revision ACLR [28]. Alessio-Mazzola et al. reported very good knee function and stability with low failure rates of 8% for revision ACLR and additional LET in 24 professional soccer players after a mean follow-up of 42 months [29]. All these studies used instrumental knee laxity measurements (KT 1000) to evaluate postoperative outcome. This presenting study did not only used instrumental measurement for postoperative but also for preoperative classification of anterior knee laxity. It is likely that Redler et al., Ferretti et al. and Alessio-Mazzola et al. included both high-grade and low-grade laxity patients in their study populations [8, 28, 29].

However, the results of this study indicate that patients without preoperative high-grade anterior knee laxity and revision ACLR do not benefit from an additional LET.

To date, there are only four studies that directly compare revision ACLR with and without LET [9, 15, 19, 30]. Trojani et al. reported the results of a retrospective multicentre study of 163 patients with revision ACLR, 51% of whom had an additional LET [19]. In a retrospective study, Ventura et al. evaluated 24 revision ACLR patients after a follow-up of 4.5 years, 12 of them were treated with and 12 without an additional LET [30]. Porter et al. compared revision ACLR in 20 patients with a pivot-shift grade 0–1 without additional LET and 18 patients with a pivot-shift grade ≥ 2 with additional LET [9].

Trojani et al., Ventura et al. and Porter et al. showed that revision ACLR combined with an additional LET led to significantly better knee stability, but differences in functional knee scores could not be demonstrated [9, 19, 30]. Alm et al. published a cohort study of 73 patients with a mean follow-up of 26 months in which 59 patients received an additional LET [15]. The total failure rate of revision ACLR was 8%. Only Alm et al. used instrumental laxity measurements to classify preoperative knee laxity. In case of preoperative high-grade anterior knee laxity (defined as SSD ≥ 6 mm), revision ACLR without an additional LET was associated with significantly higher failure rates and worse knee function compared to revision ACLR with an additional LET. In this presenting study, an additional LET did not improve knee function, stability or failure rates for patients with preoperative low-grade anterior knee laxity.

The reason for the non-superiority of revision ACLR with an additional LET in terms of knee function in the studies by Trojani et al., Ventura et al. and Porter et al. could be that the studies involved a mixed study population of patients with low-grade and high-grade anterior knee laxity.

So far, only biomechanical studies have shown that isolated ACL ruptures do not benefit from an additional LET [6]. Biomechanical studies have shown that an additional insufficiency of the anterolateral structures increases the load on the ACLR graft [31, 32]. Low-grade anterior knee laxity could indicate intact anterolateral structures and this could explain the lack of effect of an additional LET in low-grade anterior knee laxity. To the best of our knowledge, the effects of LET in the case of preoperative low-grade anterior knee laxity have not been investigated, yet.

The authors of this study believe that the extent of preoperative anterior knee laxity is an important, previously underestimated factor for revision ACLR failure. Magnussen et al. showed for primary ACLR and Alm et al. for revision ACLR that preoperative high-grade anterior knee laxity is an independent risk factor and that an additional LET may reduce the risk of ACLR failure [7, 33]. In contrast to these studies, results of this presenting study suggest that in low-grade anterior knee laxity an additional LET provides no additional benefit. The extent of preoperative anterior knee laxity may be a more important factor in the indication for an additional LET than whether it is primary or revision ACLR.

The subgroup analysis performed in this study showed that even in preoperative pivot- shift ≥ grade 2 and low-grade knee laxity, the additional LET had no statistical effect. This suggests that the degree of anterior knee laxity may be a more important factor than the pivot-shift. Ahn et al. also did not detect preoperative pivot shift ≥ grade 2 as a risk factor for ACLR failure in primary ACLR [34].

This study indicates that patients with revision ACLR without preoperative high-grade knee laxity, therefore likely without a lesion of the anterolateral structures, may not benefit from an additional LET during revision surgery. Beyond that, this study showed that the additional LET in patients with preoperative low-grade knee laxity did not influence the failure rate or postoperative functional scores. Furthermore, it was demonstrated that 17.4% of the patients suffer from pain in the area of the tenodesis which may be associated with an overconstraint of the tibial rotation in the anterolateral structures- intact knee.

While patients with preoperative high-grade knee instability, pivoting sports and general joint hyperlaxity may benefit from an additional lateral extra-articular procedure in ACLR, patients without these risk factors may be treated sufficiently with an isolated ACLR without an adjunctive LET [10, 12, 16].

There are some limitations in this study. The follow-up period was limited and postoperative long-term complications like osteoarthritis could not be observed in the 2-year follow-up. Because of the retrospective design of the study, no preoperative randomization was performed and the study size was relatively small, especially the combined LET and revision ACLR subgroup. There were more ACLR performed with hamstring autograft in the LET group (39%) than in the control group (23%) which may be a confounding factor. However, to the best of our knowledge this is the first study on the effect of the LET in low-risk patients with revision ACLR so far.

## Conclusions

Additional LET in patients with revision ACLR without high-grade anterior knee laxity does not influence patient-related outcomes or failure rates. Subjects with preoperative low-grade anterior knee laxity may not benefit from an additional LET in revision ACLR.
